# A Dual Four-Quadrant Photodetector Based on Near-Infrared Enhanced Nanometer Black Silicon

**DOI:** 10.1186/s11671-021-03499-x

**Published:** 2021-02-27

**Authors:** Guanyu Mi, Jian Lv, Longcheng Que, Yi Zhang, Yun Zhou, Zhongyuan Liu

**Affiliations:** 1 State Key Laboratory of Electronic Thin Film and Integrated Devices, University of Electronic Science and Technology of China, Cheng du, 610054 China; 2 China Electronics Technology Group Corporation, Chongqing Acoustic Optic Electronic CO.,LT, 401332, Chong qing, China

**Keywords:** Black silicon, Near infrared enhanced, Dual four-quadrant, Photodetector

## Abstract

In this paper, a new preparation process of nanometer black silicon is proposed, by which high trapping optical Se-doped black silicon material is prepared by nanosecond pulsed laser ablation of high-resistance silicon coated with Se film in HF gas atmosphere. The results indicate that the average absorptivity of 400–2200 nm band before annealing is 96.81%, and the absorptivity maintains at 81.28% after annealing at 600 degrees. Meanwhile, black silicon prepared under the new technology is used in double four-quadrant photodetector, the results show that, at a reversed bias of 50 V, the average unit responsiveness is 0.528 A/W at 1060 nm and 0.102 A/W at 1180 nm, and the average dark current is 2 nA at inner quadrants and 8 nA at outer quadrants. The dual four-quadrant photodetector based on near-infrared enhanced black silicon has the advantages of high responsiveness, low dark current, fast response and low crosstalk, hence it is appropriate for a series of direction of applications, such as night vision detection and medical field.

## Introduction

Near-infrared enhanced photodetector [1–3] is difficult to obtain satisfying performance compared with photodetectors at other wavelengths [4–6] because it is limited by response range, response rate, dark current and crosstalk in the near-infrared band. However, since Carey developed the first black silicon infrared detector in 2005, the near-infrared photodetector based on black silicon materials began to develop rapidly. The performance of black silicon developed by Carey far exceeds the performance of monocrystalline silicon infrared detector. Before long, some researchers added passivation technology to black silicon detector to reduce its dark current. Black silicon [7–9] became the preferred material for silicon-based near-infrared enhanced photodetector due to its high absorption rate and wide absorption spectrum.

As one of the most important materials in semiconductor industry, it is crucial to well manage the processing quality of black silicon materials [10–14]. The preparation of black silicon with wide spectrum, high absorption and low defect is essential for high performance near-infrared photodetector. There are some researches about preparation of black silicon materials by using femtosecond laser [15, 16] scanning at SF6 atmosphere [17, 18], and the black silicon material in the ultraviolet to near-infrared band can achieve more than 90% absorption [19]. However, the absorption in the near-infrared region is reduced to around 50% after high-temperature annealing. Meanwhile, researchers found that the absorption of Se- and Te-doped black silicon is significantly reduced by annealing compared with S-doped black silicon, but under the doping process of solid Se and Te membrane, the black silicon material is prepared in the shape of hill, and the light trapping is not good enough [20, 21].

In this paper, a new preparation process of nanometer black silicon is proposed, by which high trapping optical Se-doped black silicon material is prepared by nanosecond pulsed laser ablation of high-resistance silicon coated with Se film in HF gas atmosphere. The results indicate that the average absorptivity of 400–2200 nm band before annealing is 96.81%, and the absorptivity maintains at 81.28% after annealing at 600 degrees. Meanwhile, black silicon prepared under the new technology is used in double four-quadrant photodetector, the results show that the average unit responsiveness is 0.528 A/W at 1060 nm and 0.102 A/W at 1180 nm at a bias of 50 V, and the average dark current is 2 nA at inner quadrants and 8 nA at outer quadrants. The dual four-quadrant photodetector based on near-infrared enhanced black silicon has the advantages of high responsiveness, low dark current, fast response and low crosstalk, hence it is appropriate for a series of direction of applications, such as night vision detection and medical field.

## Method

**Fig. 1:**
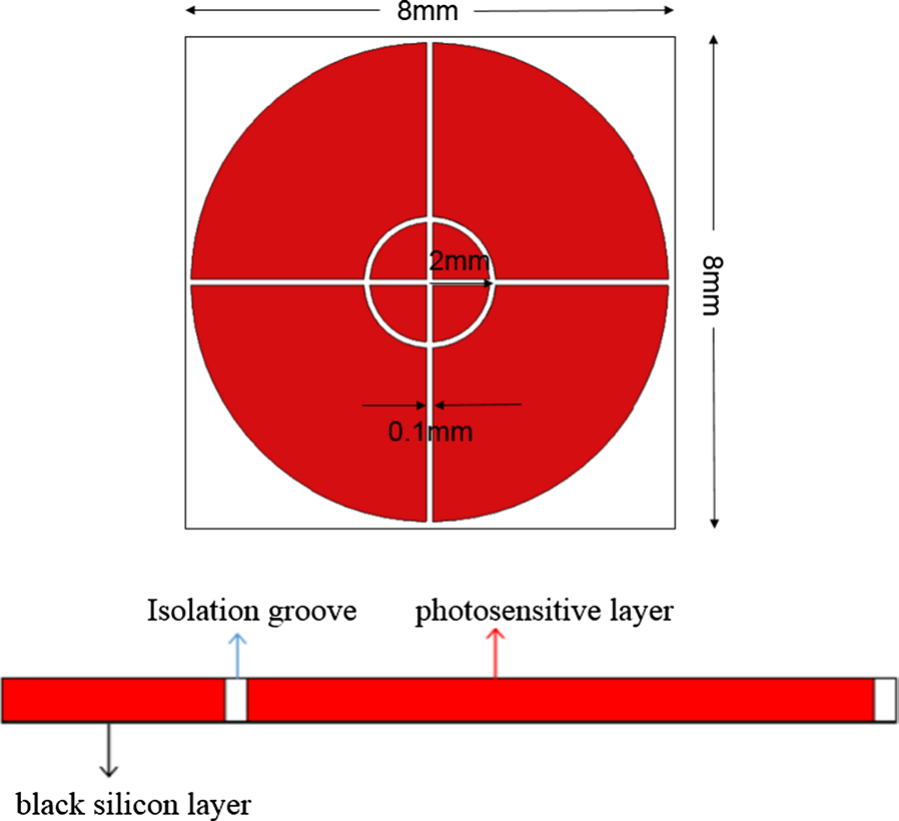
The schematic structure of dual four-quadrant photodetector

**Fig. 2:**
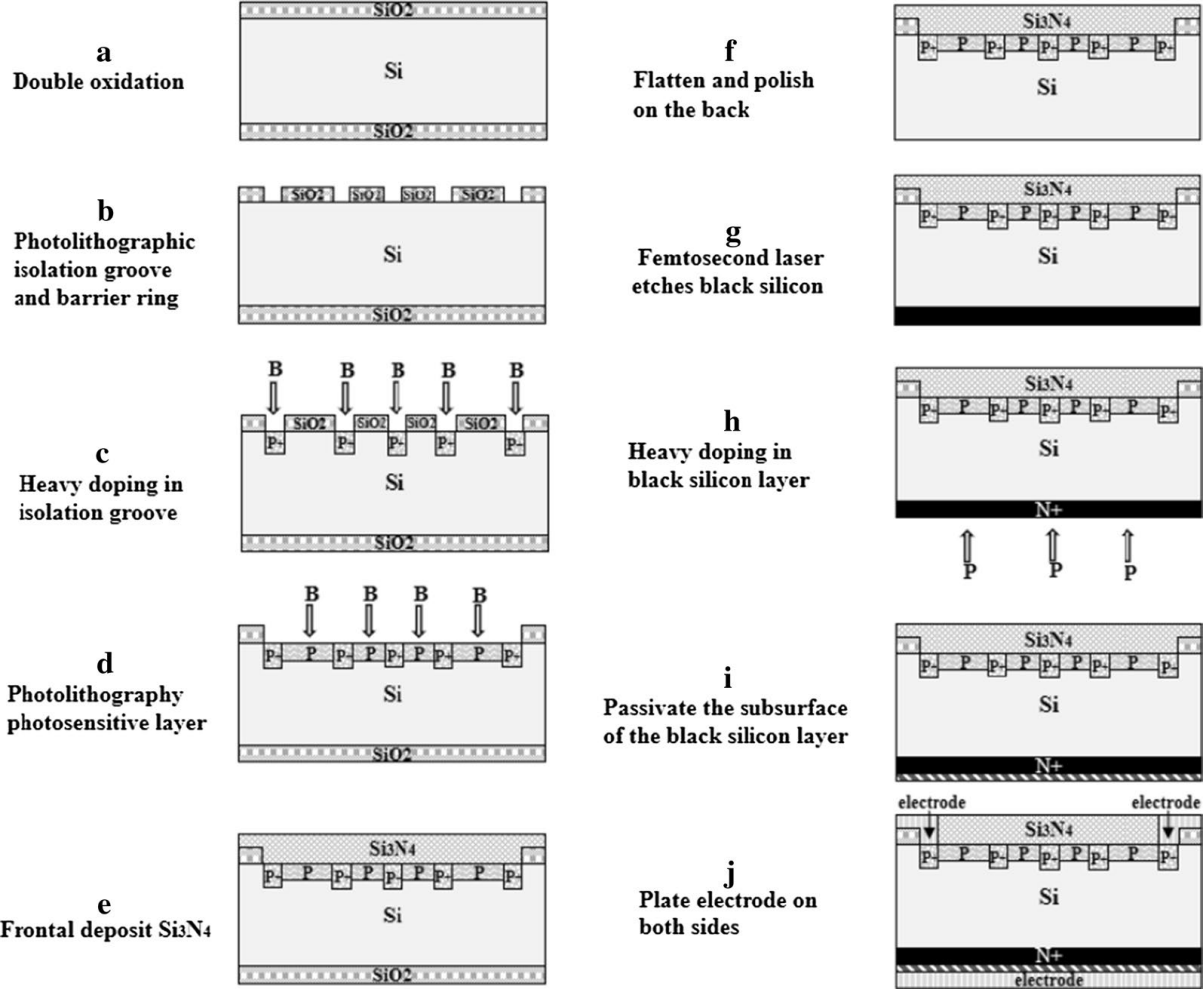
The specific manufacturing process of the photodetector

The photodetector was fabricated and tested by following processes. First, the black silicon material was prepared, N-type high-resistance silicon wafer was cut into 5 cm × 5 cm sample, and the sample was cleaned with standard cleaning procedure and blew dry in nitrogen atmosphere. Then, Se powder with 99.99% purity was used as the evaporation source, and a Se film was deposited on the surface of Si sample by vacuum coating machine. HF gas was introduced in the femtosecond laser etching process, and the processing parameters are as follows: scanning speed: 1 mm/s; laser power density: 4.5 kJ/m^2^; HF gas pressure: 9 × 10^4^ Pa. The femtosecond laser used in this paper is the Ti:sapphire femtosecond laser amplifier produced by Spectra-Physics Corporation. Second, double four-quadrant photodetector was prepared by using black silicon material, the schematic structure of dual four-quadrant photodetector and the specific manufacturing processes are shown in Figs. 1 and 2. Last, the morphologies of black silicon were characterized by a field emission scanning electron microscope (SEM), and the spectral characteristics of the material were tested by NIR2500 fiber optic spectrometer and integrating sphere. Meanwhile, the response current, dark current characteristic, rising time of photodetector were tested. During the test, the light source is a laser of the Amonics band, the dark current is measured by adding a black box to the detector to measure the current under the reversed bias, and the response time is measured by reading the change of photocurrent through an oscilloscope when using a laser pulse signal acting on detector.

## Results and Discussion

In this paper, high trapping optical Se-doped black silicon material is prepared by nanosecond pulsed laser ablation of high-resistance silicon coated with Se film in HF gas atmosphere. On the one hand, the effect of annealing on black silicon is reduced because the Se coating is supersaturated instead of using the traditional S-doped silicon. The diffusion rate of S atoms out Si lattice is faster than Se; therefore, the annealing effect is poor. On the other hand, HF is decomposed into H+ and F− at high temperature, and F ion interacts with silicon material ablated by femtosecond laser at high temperature to produce volatile SiF4; in this way, the surface of the material is continuously etched, forming a nanoscale pyramid structure, the nanoscale pyramid produced by laser etching effectively reduces the reflectivity of black silicon. Meanwhile, surface passivation optimizes the lifetime of the minority carriers, and it reduces the defect density of black silicon material and unnecessary carrier recombination. Femtosecond laser etching is simple and reproducible, by which the uniformity of the black silicon array is good, while the black silicon bandgap width can be greatly reduced. By further studying the influence of gas atmosphere, laser power and laser scanning speed on the properties of black silicon material, the optimized process flow can be obtained. The black silicon has a significant improvement in absorption after annealing prepared by the new process.

Dual four-quadrant photodetector is manufactured by using black silicon material under the new process; the schematic structure proposed in this paper is illustrated in Fig. 1. The photodetector proposed is composed of photosensitive layer, isolation groove and black silicon layer. The outer diameter of the photosensitive surface is 8 mm, while the inner diameter is 2 mm, and the photosensitive areas are separated from each other by isolation slots. The proposed photodetector can determine the offset size and orientation of the target relative to the optical axis according to different quadrant detection results, thus achieving accurate positioning.

The response current, dark current characteristic, rising time and crosstalk characteristic of the photodetector are simulated by commercial software COMSOL Multiphysics 5.4a in order to design the optimal structure. The response current, dark current characteristic, rising time of photodetector can be obtained by Eqs. 1–3. It can be seen that the response current, dark current and response time are closely related to the thickness of layer I and bias voltage when the area, incident power and material parameter are determined; therefore, these parameters are mainly simulated.

1 $${\text{I}}_{{\text{p}}} = \frac{{qP\left( {1 - R} \right)}}{hv} \cdot \left( {1 - \frac{{e^{ - \alpha W} }}{{1 + \alpha \sqrt {D\tau } }}} \right) + qP\frac{D}{{\sqrt {D\tau } }}$$

2 $${\text{I}}_{D} = \sqrt {Aqn\frac{W}{2\tau }} + \left( {\frac{2m}{{E_{g} }}} \right)^{\frac{1}{2}} \left( {q^{3} E\frac{v}{{4\pi^{2} \hbar^{2} }}} \right)Ae^{{\left( { - \frac{4}{3qE\hbar }\sqrt {2mE_{g}^{3} } } \right)}}$$

3 $$T = \sqrt {\left( {2.2t_{RC} } \right)^{2} + t_{d}^{2} + \tau_{d}^{2} }$$

In which P stands for incident power, R is reflectance, α is absorption coefficient, W stands for the thickness of layer I, D is hole diffusion coefficient, and τ is Carrier life. E $\propto$ bias voltage, t_RC_ stands for circuit time constant which is mainly determined by the equivalent resistance and capacitance. t_d_ is diffusion time, and τ_d_ is transit time.

**Fig. 3:**
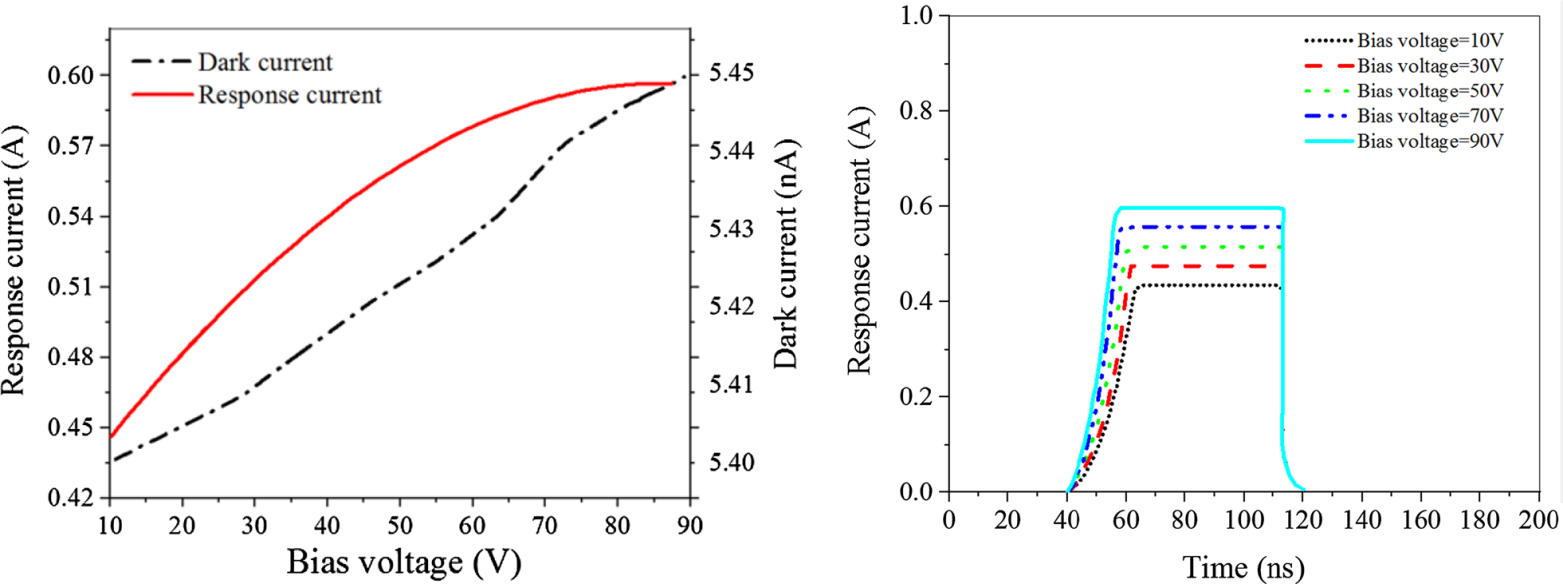
The response current, dark current characteristic and rising time change curve of photodetector at different reversed bias voltage

**Fig. 4:**
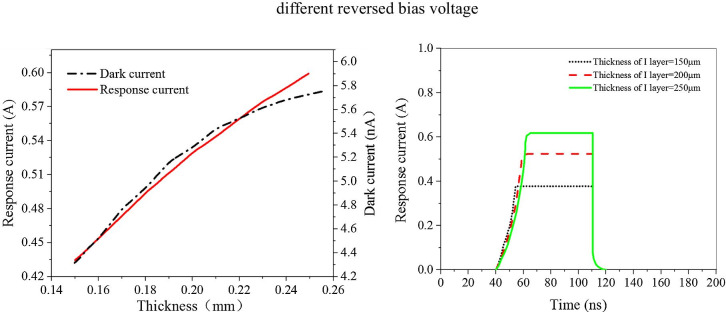
The response current, dark current characteristic and rising time change curve of photodetector at different thickness of layer I

**Fig. 5:**
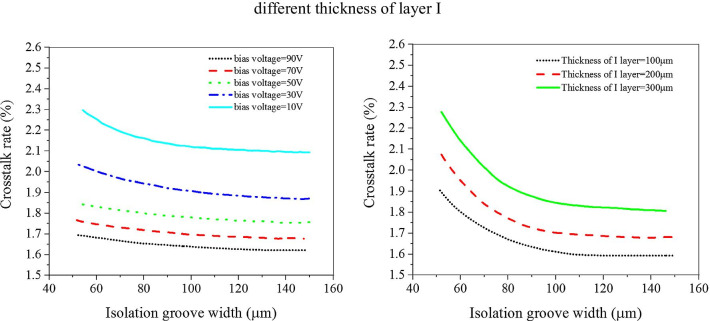
The influence of isolation slot width on crosstalk rate

The influences of reversed bias voltage on the above parameters are illustrated in Fig. 3, it can be seen that with the increases in the bias voltage, the response current and the dark current will be increased as well; however, the rising time will be decreased. Therefore, it is necessary to balance the contradiction between response current, rising time and dark current as the bias increases and to choose the appropriate bias according to the demand. In the same way, the thickness of layer I of the PIN structure, which greatly determines the thickness of the photodetector, is also simulated and the results are shown in Fig. 4. Meanwhile, Fig. 5 gives influence of isolation slot width on photodetector, it can be seen that when the isolation slot width is increased to 100 μm, the crosstalk rate is basically stable. According to the simulation results, the optimal response current, dark current and rising time are obtained, the specific device parameters are shown in Table 1.

**Table 1:** The geometry parameters of structure

Outer diameter	Inner diameter	Thickness	Reversed bias voltage	Isolation slot width
8 mm	2 mm	0.2 mm	50 V	0.1 mm

Response current	Dark current	Rising time
0.53 A at 1060 nm	5.2 nA	12 ns

In order to achieve high response, fast response speed and high stability of the photodetector, some manufacturing processes also have been optimized [22–24]. First, the isolation groove and blocking ring are designed to reduce the crosstalk between adjacent photosensitive areas. Second, wafer thinning and polishing processes are used to reduce depletion layer thickness to improve device response speed. Third, the preparation of black silicon by one-step femtosecond laser ablation is the crucial to achieve good repeatability and stability of black silicon materials. Last, the subsurface passivation treatment of black silicon layer is used to reduce and regulate the density of surface defect state and reduce the deadweight compound of photogenic carriers to achieve high responsiveness of the photodetector. The specific manufacturing process of the photodetector is shown in Fig. 2. The final device diagram is shown in Fig. 2j, in which the thickness of layer I is 180 μm and the thickness of layer P N is 10 μm, P^+^ is formed by heavy doping of B on P type silicon, N^+^ is formed by diffusion of P, and the contact electrode was deposited by thermal evaporation.

**Fig.6:**
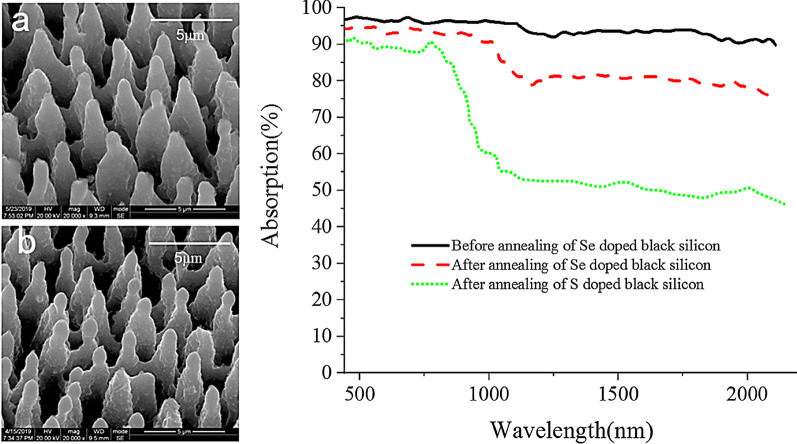
The changes of the surface morphology and photoelectrical properties of material after high-temperature annealing

**Fig. 7:**
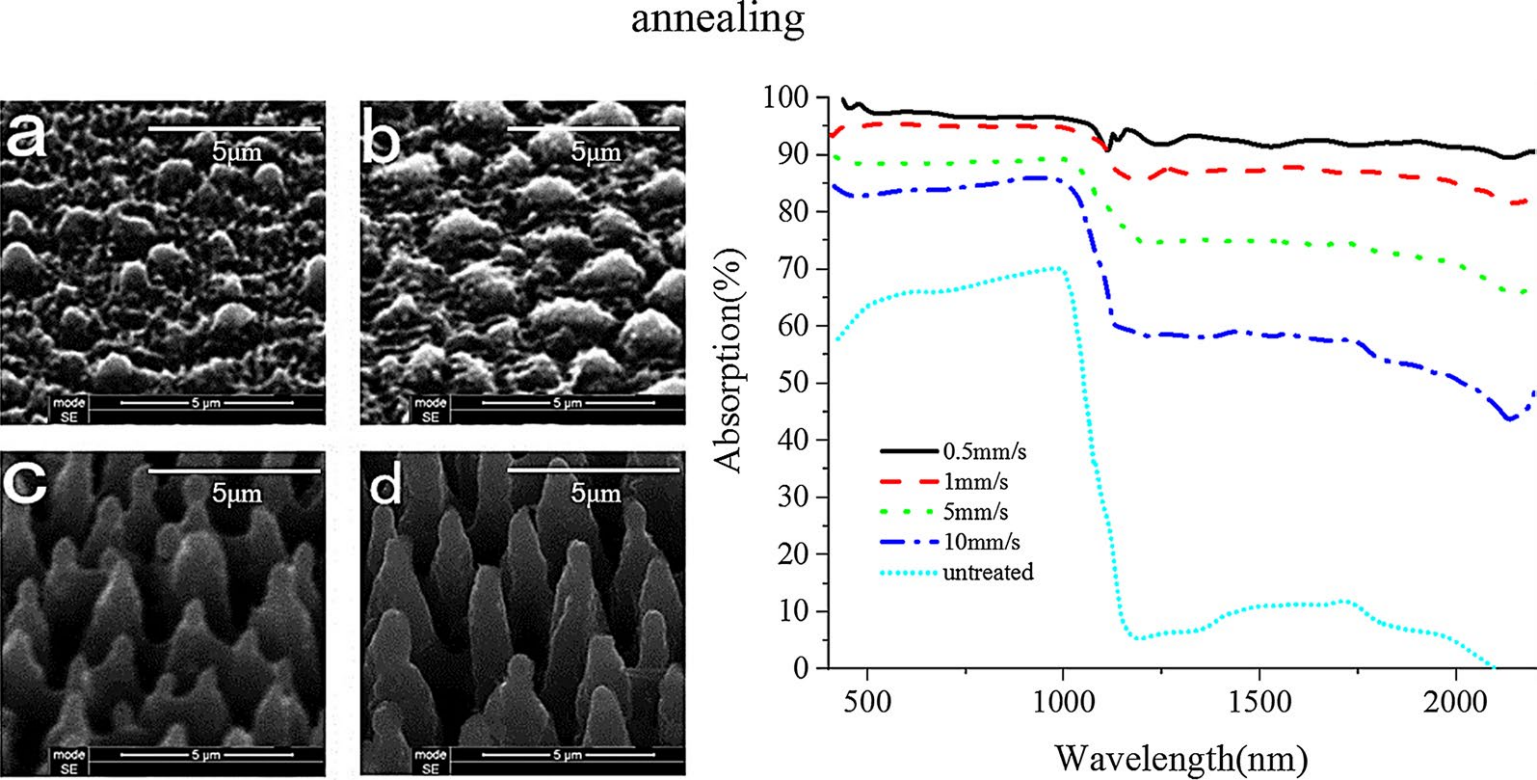
The surface morphology and absorption spectra of the materials at different scanning speeds **a** 10 mm/s, **b** 5 mm/s, **c** 2 mm/s, **d** 1 mm/s

Figure 6 shows the changes of the surface morphology and photoelectrical properties of high-notch light-sensitive Se-doped black silicon after high-temperature annealing, the specific machining parameters are as follows: scanning speed: 1 mm/s; laser power density: 4.5 kJ/m^2^; HF gas pressure: 9 × 10^4^ Pa. It can be seen in figure that the surface morphology before and after high-temperature annealing is more evenly distributed on the nanoscale tapered black silicon array without obvious change. In terms of the absorption spectrum, the average absorption rate after annealing of black silicon made under the new process in this paper reached 83.12%, the fire resistance improved significantly compared with the absorption rate of about 50% after annealing of S-doped black silicon. Furthermore, the effect of femtosecond laser pulse scanning speed on the performance of black silicon material was tested, and the results are illustrated in Fig. 7. It can be seen that with the decrease in velocity, the doping amount of Se element increases continuously, which leads to the more obvious shape of black silicon tip cone and higher absorption rate.

According to Tauc mapping theory, the bandgap of material can be obtained by the transformation of its absorption spectrum [25]:

4 $${\text{F}}\left( {{\text{R}}\infty } \right) \approx \frac{{{\text{A}}^{{2}} }}{{{\text{2R}}}}$$

5 $$\left( {{\text{h}}\nu \alpha } \right)^{{\frac{{1}}{{\text{n}}}}} = {\text{K}}\left( {{\text{h}}\nu - {\text{Eg}}} \right)$$

6 $${\text{h}}\nu = \frac{{{1239}{\text{.7}}}}{\lambda }$$

7 $$\left( {{\text{h}}\nu {\text{F}}\left( {{\text{R}}\infty } \right)} \right)^{{\frac{{1}}{{2}}}} = {\text{K}}\left( {{\text{h}}\nu - {\text{Eg}}} \right)$$

In which A stands for spectral absorption, R is reflectance. The inflection point (the maximum point of the first derivative) is obtained by calculating the first derivative of the hv-(hvF(R∞))^1/2^ curve, and the tangent of the curve is made at this point. The abscissa value of the intersection of the tangent and the X axis are the bandgap of the sample. The equivalent bandgap width results of black silicon materials at different scanning speeds are shown in Table 2, with the decrease in scanning speed and the increase in Se doping concentration, the bandgap width is decreasing compared with the 1.12 eV of traditional silicon materials, and the spectral band is increasing.

**Table 2:** The bandgap changes of scanning speed

Scanning speed	0.5 mm/s	1 mm/s	5 mm/s	10 mm/s	Untreaded
Bandgap	1.02 eV	1.05 eV	1.08 eV	1.10 eV	1.12 eV

**Fig.8:**
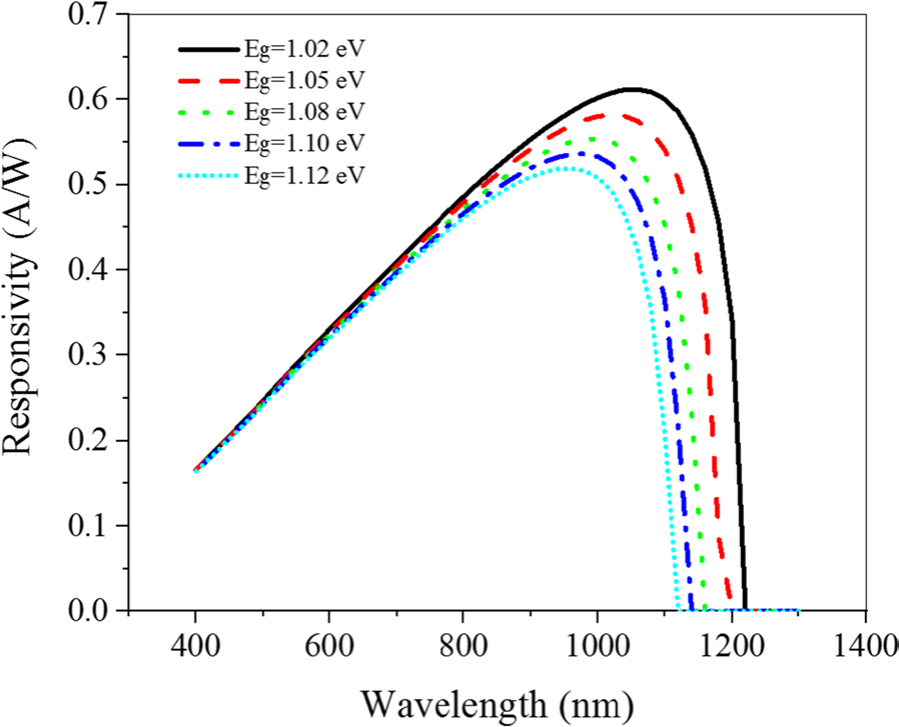
Responsivity of black silicon by different bandgap

The PIN junction of dual four-quadrant photodetector is simulated at different bandgap of materials. The simulation results are illustrated in Fig. 8; the results show that with the decrease in the bandgap width, photocurrent absorption peak is shifted towards the near-infrared band. Therefore, considering the simulation results, the optical and electrical performance of the photodetector, the optimal scanning speed can be selected.

**Fig. 9:**
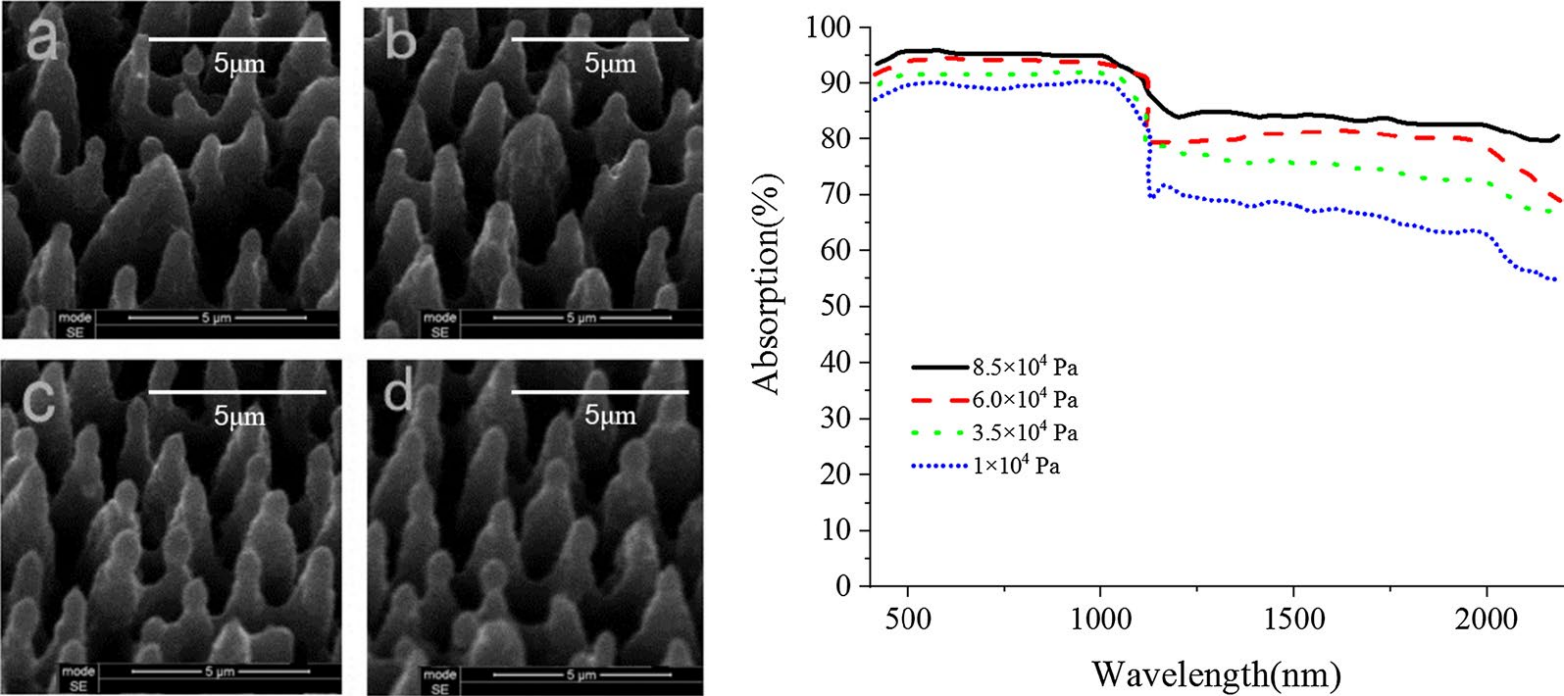
The surface morphology and absorption spectra of the materials at different HF air pressure **a** 1 × 10^4^ Pa, **b** 3.5 × 10^4^ Pa, **c** 6 × 10^4^ Pa, **d** 8.5 × 10^4^ Pa

**Fig. 10:**
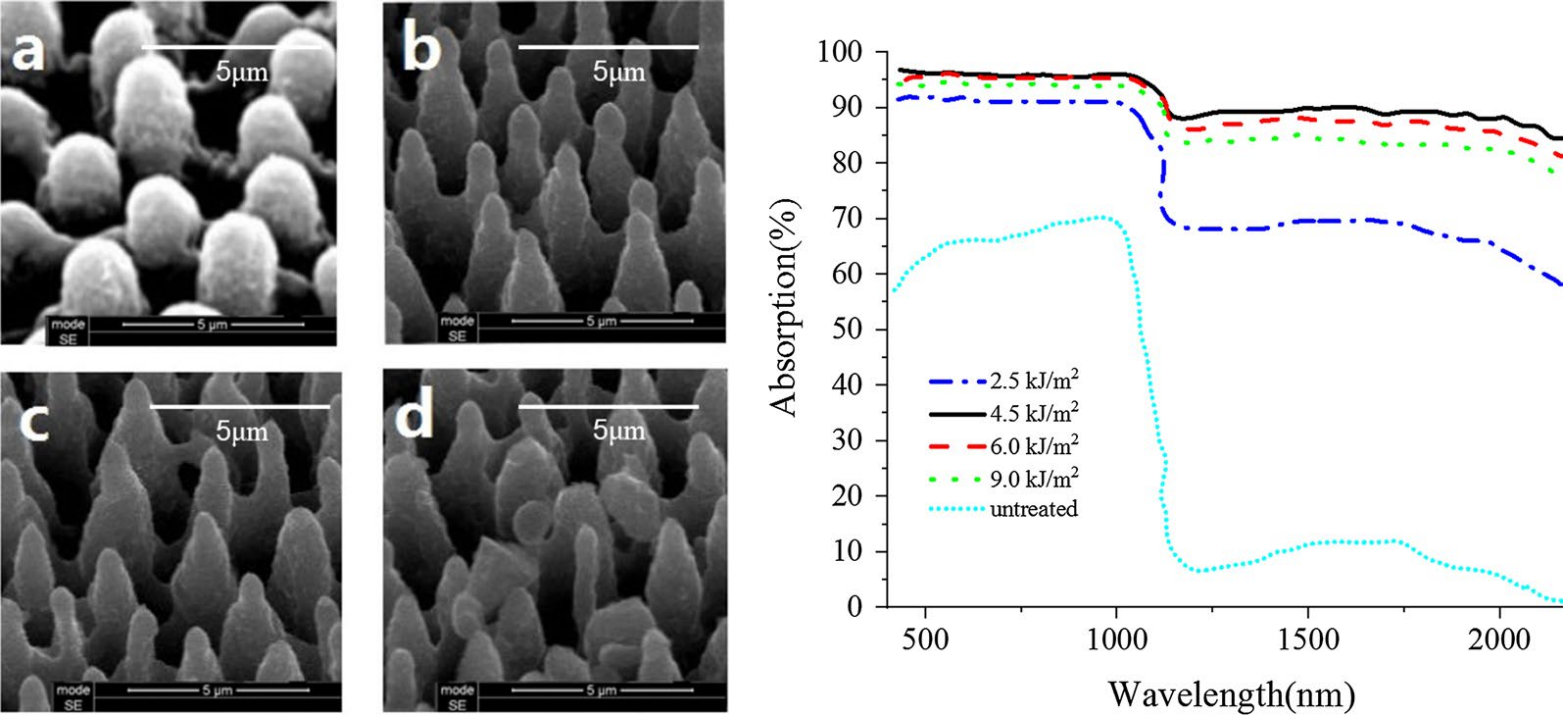
The surface morphology and absorption spectra of the materials at different optical power density **a** 2.5 kJ/m^2^, **b** 4.5 kJ/m^2^, **c** 6.0 kJ/m^2^, **d** 9.0 kJ/m^2^

The same simulation process is used to determine the optimal material preparation parameters under different experimental conditions, such as optical power density and HF air pressure, which are shown in Figs. 9 and 10.

**Fig. 11:**
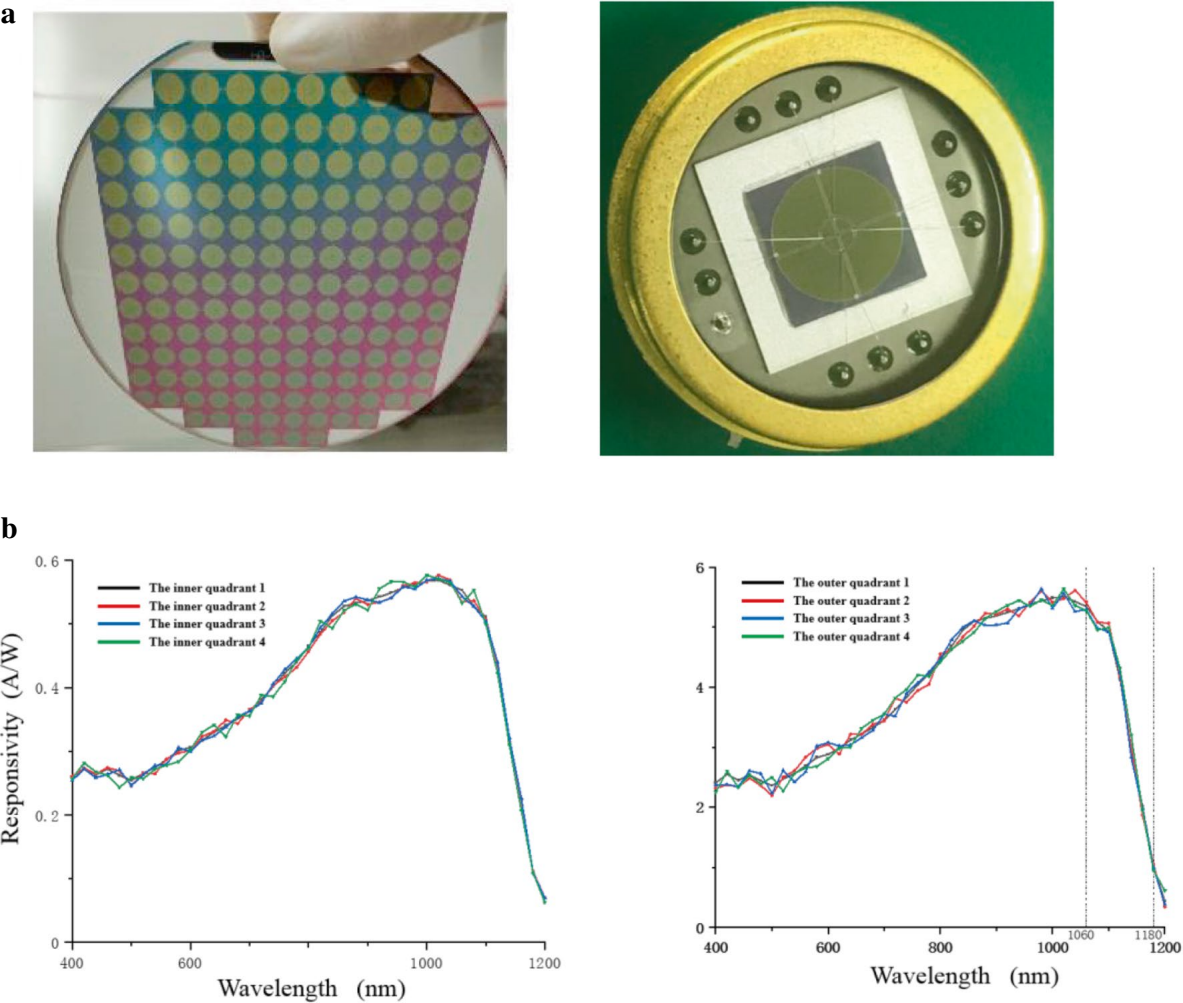
a The physical picture of dual four-quadrant photodetector. **b** The responsivity of different dual four-quadrant photodetector samples

The specific machining parameters are as follows: scanning speed: 1 mm/s; laser power density: 4.5 kJ/m^2^; HF gas pressure: 9 × 10^4^ Pa, under the above experimental parameters, the black silicon material was prepared by the new technology, and the double four-quadrant photodetector was made. The physical picture of the photodetector and the test results are shown in the Fig. 11, Tables 3 and 4, and the results of responsiveness are measured by layer of 2 mW. The results show that the average unit responsiveness is 0.528 A/W at 1060 nm and 0.102 A/W at 1180 nm at a reversed bias of 50 V, the response band ranges from 400 to 1200 nm, which are basically the same as the simulation result. The average spectral absorption rate is over 90%, and the average dark current is less than 8 nA, the dark current is measured by adding a black box to the detector to measure the current under the reversed bias, and the results of dark current are a little larger than the simulation results, because the depth uniformity of the junction in the photosensitive region is not ideal in the actual processing. Meanwhile, the response time is measured by reading the change of photocurrent through an oscilloscope when using a laser pulse signal acting on detector, and the average rising time is less than 12 ns, which conforms the expected simulation results. Therefore, the photodetector manufactured in this paper not only achieves four-quadrant precise positioning, but also ensures wide detecting band, low dark current and fast response.

**Table 3:** Test results of dark current

Quadrant		Dark current (nA)	Quadrant		Dark current (nA)
Outer quadrant	1	8	Inner quadrant	1	2
	2	8		2	2
	3	8		3	3
	4	7		4	2

**Table 4:** Test results of response speed

Quadrant		Rising time (ns)	Quadrant		Rising time (ns)
Outer quadrant	1	11	Inner quadrant	1	11
	2	11		2	10
	3	12		3	12
	4	12		4	11

## Conclusions

In this paper, a new preparation process of black silicon is proposed, by which high trapping optical Se-doped black silicon material is prepared by femtosecond laser ablation of high-resistance silicon coated with Se film in HF gas atmosphere. The results indicate that the average absorptivity of 400–2200 nm band before annealing is 96.81%, and the absorptivity maintains at 81.28% after annealing at 600 degrees. Meanwhile, black silicon prepared under the new technology is used in double four-quadrant photodetector, the results show that the average unit responsiveness is 0.528 A/W at 1060 nm and 0.102 A/W at 1180 nm at a bias of 50 V, and the average dark current is 2 nA at inner quadrants and 8 nA at outer quadrants. The dual four-quadrant photodetector based on near-infrared enhanced black silicon has the advantages of high responsiveness, low dark current, fast response and low crosstalk, hence it is appropriate for a series of direction of applications, such as night vision detection and medical field.

## Data Availability

The datasets used or analyzed during the current study are available from the corresponding author on reasonable request.

## Abbreviations

SEM: Scanning electron microscopy
NIR: Near infrared
